# The 1027^th^ target candidate in stroke: Will NADPH oxidase hold up?

**DOI:** 10.1186/2040-7378-4-11

**Published:** 2012-05-24

**Authors:** Kim A Radermacher, Kirstin Wingler, Pamela Kleikers, Sebastian Altenhöfer, Johannes JR Hermans, Christoph Kleinschnitz, Harald HHW Schmidt

**Affiliations:** 1 Department of Pharmacology & Cardiovascular Research Institute Maastricht (CARIM), Maastricht University, Maastricht, The Netherlands; 2 Neurologische Klinik und Poliklinik, Universität Würzburg, Würzburg, Germany

**Keywords:** NADPH oxidases (NOX), Oxidative stress, Stroke therapy

## Abstract

As recently reviewed, 1026 neuroprotective drug candidates in stroke research have all failed on their road towards validation and clinical translation, reasons being quality issues in preclinical research and publication bias. Quality control guidelines for preclinical stroke studies have now been established. However, sufficient understanding of the underlying mechanisms of neuronal death after stroke that could be possibly translated into new therapies is lacking. One exception is the hypothesis that cellular death is mediated by oxidative stress. Oxidative stress is defined as an excess of reactive oxygen species (ROS) derived from different possible enzymatic sources. Among these, NADPH oxidases (NOX1-5) stand out as they represent the only known enzyme family that has no other function than to produce ROS. Based on data from different NOX knockout mouse models in ischemic stroke, the most relevant isoform appears to be NOX4. Here we discuss the state-of-the-art of this target with respect to stroke and open questions that need to be addressed on the path towards clinical translation.

## Lack of mechanistic insight hampers innovation

One third of all patients do not survive a stroke, and in those who do, brain damage can be so severe that it results in life-long disability [1]. In this review, we will focus on ischemic stroke (87% of all stroke cases) and on both, the disturbed oxygen and nutrient supply to the infarcted region and the consequences of reperfusion.

Neurons start dying already 5 minutes after lacking oxygen. The only approved treatment for acute stroke is an early intravenous administration of recombinant tissue plasminogen activator (rt-PA) to restore cerebral blood flow. However, even if blood flow is restored and no bleeding occurs, reperfusion can paradoxically aggravate neuronal damage. Mechanistically, reactive oxygen species (ROS) generated from the newly arriving oxygen, i.e. ischemia-reperfusion (I/R) injury, are thought to play a major role in this. Hence, to prevent brain damage after an ischemic stroke novel therapeutic strategies that specifically address this paradox are urgently needed.

## A roadblock in stroke research

Unfortunately, despite tremendous research activities, all therapeutic strategies that at some stage showed promising results in pre-clinical animal models have finally failed before or in clinical testing [2]. A systematic review covering 8516 stroke-relevant studies added this up to 1026 candidate strategies finally failing [3]. 114 out of 1026 neuroprotective drugs were tested in patients, 42% even quite pre-maturely, i.e. before the results from animal testing were reported. On top of this, drugs that had been selected for clinical trials were not necessarily the most efficient ones based on animal data. This roadblock in stroke innovation [4] has raised fundamental concerns about the general approach and translatability of pre-clinical stroke research. Possible explanations for an impaired translatability are the divergences between pre-clinical animal models and the clinical situation in real patients [5]. Therefore, older animals with co-morbidities should be tested and actual clinical endpoints should be considered in order to better mimic clinical conditions (Table 1) [6]. Furthermore, only very few studies (3%) reported sample size calculations, and thus the majority of studies might have been underpowered to detect real differences (Table 1). Another important aspect is the problem of publication bias in favour of positive results [7]. These quality concerns are not only related to the field of stroke research and rather apply to animal models in general. To improve the quality of pre-clinical stroke research the Stroke Therapy Academic Industry Roundtable (STAIR) published criteria in 1999 [8], which were updated in 2009 [9].

**Table 1:** Prevalence of pre-clinical stroke guidelines in stroke studies using NOX knockout mice

**NOX isoform**	**Parameters analysed**	**Transient MCAO model**	**Permanent model**	**Age/ Weight**	**Gender**	**Litter-mates**	**Power**	**Operator- blinded**	**>24 h**	**Outcome**	**Reference**
**NOX1**	mortality, infarct, edema, functional outcome	30 min/23.5 h	✗	11-17 wk	m	✓	n.s.	✓	✗	cortical but not total infarct ↑ in NOX1 KOs	[10]
	infarct, BBB, functional outcome, apoptosis, NOX1 mRNA + protein levels	1/23 h and 2/22 h	pMCAO	n.s.	m	✓	n.s.	n.s.	✗	NOX1 KOs protected	[11]
	mortality, infarct, edema/BBB, functional outcome, hemorraghe, NOX1 mRNA + protein levels, ROS + RNS, apoptosis.	1/23 h and 1 h/6d	pMCAO, cortical PT	6-8 wk	m	✗	93%	✓	6 d	no significant difference	[12]
**NOX2**	infarct, ROS, neutrophils	2/22 h	✗	8-10 wk	m	✓	n.s.	n.s.	✗	NOX2 KOs protected but not with WT bone marrow implant	[13]
	infarct, ROS	25 min/3d	✗	6 wk	m	✓	n.s.	n.s.	3 d	NOX2 KOs protected	[14]
	infarct, BBB	2/22 h	✗	7-9 wk	m	✗	n.s.	n.s.	✗	NOX2 KOs protected	[15]
	infarct, functional outcome, oxidative stress, cell death, neutrophils, inflammation	75 min/22.75 h and 75 min/3d	✗	12-16 wk	m	✗	n.s.	n.s.	3 d	NOX2 KOs protected	[16]
	mortality, infarct, functional outcome, ROS	30 min/23.5 h	✗	6-8 wk	m	✗	n.s.	✓	✗	NOX2 KOs protected	[17]
	infarct, edema, functional outcome, NOX2 protein levels, ROS.	30 min/23.5 h and 30 min/3d	pMCAO	6-8 wk	m + f	✗	n.s.	✓	3 d	only male NOX2 KOs protected	[18]
	mortality, infarct, edema/BBB, functional outcome, hemorraghe, mRNA + protein levels, ROS + RNS, apoptosis.	1/23 h and 1 h/6d	pMCAO, cortical PT	6-8 wk and 18–20 wk (unpublished)	m	✗	93%	✓	6 d	no significant difference	[12]
	infarct, RNS, cell death, inflammatory markers	75 min/24 h and 75 min/72 h	✗	12-16 wk	m	✗	n.s.	n.s.	3 d	NOX2 KOs protected	[19]
	infarct, BBB, functional outcome, hemorraghe, ROS	2/22 h	✗	25-30 g	m	✗	n.s.	n.s.	✗	NOX2 KOs protected	[20]
	mortality, infarct, edema, functional outcome, NOX2 protein levels, ROS + RNS, NO function.	30 min/23.5 h	✗	8-12 wk	m	✗	n.s.	✓	✗	NOX2 KOs protected	[21]
**NOX4**	mortality, infarct, edema/BBB, functional outcome, hemorraghe, mRNA + protein levels, ROS + RNS, apoptosis.	1/23 h and 1 h/6d	pMCAO, cortical PT	6-8 wk and 18–20 wk	m + f	✗	93%	✓	6 d	NOX4 KOs protected	[12]

Published studies evaluating the role of NADPH oxidases in stroke by means of KO mice were assessed for their translatability. Parameters that should be investigated when performing a stroke study are, amongst others, the use of aged mice of both genders, and wildtype littermates as negative controls. In addition to transient models, a permanent model should be added, and animals should be assessed for longer time periods than 24 h. Finally, power calculations should be reported. All of the above cited studies used temperature control and monitored cerebral blood flow. Unfortunately, none of these studies included animals that present co-morbidities.

BBB: blood brain barrier leakage; d: days; f: female; h: hours; KO: knock out; m: male; min: minutes; n.s.: not specified; pMCAO: permanent middle cerebral artery occlusion; PT: photothrombosis; ROS: reactive oxygen species; RNS: reactive nitrogen species; wk: weeks.

## Mechanism of stroke #1027: Oxidative stress and how to tackle it

How was the oxidative stress hypothesis addressed so far and why do we believe that NADPH oxidase is a highly promising target to induce neuroprotection?

ROS are non-radical and radical reactive molecules. They are formed when one electron is transferred to molecular oxygen, forming the superoxide anion (O_2_^-^). O_2_^-^ can be rapidly dismutated into more stable species such as hydrogen peroxide (H_2_O_2_), or - in the presence of nitric oxide (NO) - it forms the peroxynitrite anion (ONOO^-^). Importantly, all of these form a potentially disease-triggering mixture of ROS.

ROS have a short half-life and are therefore difficult to measure. Furthermore, there is no method that is able to distinguish the different types of ROS formed. Often, global ROS levels are measured and localised in situ using dihydroethidium (DHE), which, after its oxidation, can be detected as red nuclear fluorescence [22]. In vivo, measuring ROS using DHE is difficult because the intravenously injected DHE hardly reaches ischemic regions [11].

Importantly, ROS are not only detrimental. Rather, they also serve as essential signalling molecules that for example regulate vascular tone, oxygen tension and erythropoietin production [23]. In a healthy brain, as in any organ, there is a balance of pro- and anti-oxidants. If pro-oxidants become too prominent, oxidative stress arises. Most times, oxidative stress is caused by an elevated production of ROS. This may be an important underlying mechanism of I/R damage caused by an ischemic stroke.

The free-radical scavenger NXY-059 was first considered as a major breakthrough by stroke researchers. The drug fulfilled all major STAIR criteria and its highly promising pre-clinical data, even in non-human primates, led to the largest neuroprotective trials in stroke research. Whereas the first, although underpowered, phase III trial (SAINT I) still left room for hope by improving disability 90 days post stroke after NXY-059 treatment [24], a second larger trial (SAINT II) highlighted the inefficiency of the anti-oxidative drug [25]. After the huge disappointment, many scientists were preoccupied with the question why this promising radical scavenger finally failed? Reasons may be that different outcomes have been measured and that there was a treatment delay compared to pre-clinical studies [26]. NXY-059 is also a weak antioxidant and has poor blood–brain-barrier penetration [27]. Bath et al. [28] also suggested that negative publication bias (unpublished data from AstraZeneca) might have led to an initial overestimation of efficacy.

In general, all major clinical trials aiming to provide proof-of-principle of the oxidative stress theory by applying antioxidants have failed or can even be harmful and lead to increased mortality [29,30]. Nevertheless, it would be a mistake to dismiss the oxidative stress hypothesis.

Many likely reasons for the lack of clinical efficacy of antioxidants, also for other diseases have been suggested, which are discussed in more detail elsewhere [31,32]. The possible reasons, for example, include differences between animal models and patients as discussed above, e.g. presence/absence of co-morbidities, gender and age. Additionally, it is unlikely that antioxidant supplements can work in humans as for example ROS are not evenly distributed within the body, or not even within a cell, and supplemented antioxidants are not targeted to these specific sites. Further, antioxidants cannot distinguish between physiological and pathological ROS. ROS may also cause damage already before they are inactivated by antioxidants.

A far superior strategy may thus be to tackle the problem at its root by inhibiting the formation of ROS in the first place rather than attempting to scavenge them after they have been formed [31]. However, a prerequisite is to identify the disease-relevant sources of ROS. Several sources of ROS exist, including mitochondria [33], xanthine oxidase (XO) [34,35], monoamine oxidase [36], tyrosine hydroxylase [36], L-amino acid oxidase [36], lipid peroxidases [37], uncoupled nitric oxide synthase (NOS) [38], cytochrome P450 (CYP450) [39], cyclooxygenase (COX) and NADPH oxidases (NOX) [36]. Except for one enzyme family, all these sources have primarily functions other than producing ROS. They rather form ROS as by-products (mitochondria) because of substrate/cofactor shortage (XO, NOS), or as physiological intermediates (CYP450, COX). Only NADPH oxidases have ROS production as primary role [40,41]. NADPH oxidases are a major source of ROS in the vascular system [42], in phagocytes [43], in cerebral vessels [36] and perhaps also in neurons [44].

## NADPH oxidases

NADPH oxidases are multi-subunit complexes. Seven homologues of the catalytic subunit exist, NOX1-5 and the dual oxidases DUOX1 and 2 that also contain a peroxidase-like domain [41,45,46]. NADPH oxidases not only differ in their catalytic subunit NOX, but also in their subunit requirements, in tissue and (sub)cellular localization and also in the nature of the ROS produced (Table 2) [41,46,47].

**Table 2:** Overview of regulation, ROS product, and localization of the different NOX isoforms

Isoform	Regulators	Product	Tissue distribution	Cellular distribution
NOX 1	NOX1 subunits, Rac, PDI, Hsp90, hypoxia	O_2_^-^	Brain, vessels, colon, stomach, uterus, placenta, prostate, retina.	Neurons, astrocytes, microglia, VSMCs, epithelial cells, osteoclasts,
NOX 2	NOX2 subunits, Rac, Hsp90, hypoxia	O_2_^-^	Brain, vessels, liver, muscle.	Neutrophils, monocytes, macrophages, T-cells, microglia, astrocytes, ECs, fibroblasts, cardiac myocytes, hepatocytes, hemapoietic stem cells.
NOX 4	p22^phox^, PolDip2, PDI, hypoxia	H_2_O_2_	Ubiquitous, especially kidney, vessels, lung, bone.	Neurons, astrocytes, ECs, VSMCs, fibroblasts, mesangial cells, keratinocytes, osteoclasts, hepatocytes.
NOX 5	no subunits, but calcium sensitive, Hsp90	O_2_^-^	Testis, spleen, kidney, lymphatic tissue, uterus	ECs, VSMCs, lymphocytes, and several cancer cell lines

ECs, endothelial cells; H_2_O_2_, hydrogen peroxide; Hsp90: heat shock protein 90; O_2_^-^, superoxide, PDI: protein disulphide isomerase; PolDip2: polymerase (DNA-directed) delta interacting protein; VSMCs, vascular smooth muscle cells.

### Tissue distribution and cellular localization

NOX1, 2, 4 and 5 are expressed in blood vessels. However, NOX5 is not present in rats and mice. NOX3 is expressed in the inner ear and DUOX1 and DUOX2 are expressed in thyroid, with DUOX2 also being present in lung and gastrointestinal tract epithelium. [47,48]. As NOX3, as well as DUOX1 and DUOX2, are neither expressed in the vasculature nor in the brain, they are not relevant for stroke and will thus not be further discussed in this review. A summary of the tissue and cellular distribution of NOX1, 2, 4 and 5 is given in Table 2.

NOX1 is predominantly found in colon epithelia [49], but also at lower levels in cerebral cells (neurons, astrocytes and microglia) [50] and in vessels. In the vasculature, NOX1 is usually restricted to vascular smooth muscle cells (VSMCs) [51]. Indeed, NOX1 knockout (KO) mice have slightly reduced blood pressure and a reduced pressor response to angiotensin II [52,53].

NOX2 was first discovered in phagocytes and was previously called gp91^phox^. The role of NOX2 in neutrophils is host defence [41]. Besides circulating neutrophils, it was also detected in the brain (mainly in microglia) and in vascular cells [41]. In vessels, NOX2 is present in endothelial cells (ECs) and fibroblasts [54], and also in infiltrating monocytes, macrophages and T-cells when underlying pathology is present.

NOX4 (previously called renox) is widely distributed with high physiological levels in the kidneys [55], lung [56] and vasculature [57]. Therefore, knocking out the NOX4 gene was expected to induce arteriolar hypotension, altered pulmonary and renal function. However, surprisingly, deletion of NOX4 did not cause any respective abnormal basal phenotype [12,58]. NOX4 is the most dominant isoform in vessels and its levels are even higher in cerebral vessels [59]. NOX4 mRNA is expressed in ECs, VSMCs and fibroblasts [40]. Expressed and functionally active NOX4 has also been found in neurons [60] and astrocytes [11].

As NOX5 is not expressed in rodent cells, it has not been studied in classical NOX KO studies [61]. In other species, NOX5 was detected in testis, spleen, kidney, lymphocytes, ECs and VSMCs [62].

In summary, different NOX homologues have different functions in different cell types, and subcellular localization is also an important determinant of NOX function. For more detailed information we refer to other reviews [41,47].

### Regulation

All NOX isoforms contain six trans-membrane domains with two heme-binding sites and on the cytosolic tails binding-sites for FAD and NADPH [63]. A number of regulatory subunits have been identified for the NADPH oxidases. NOX1-4 form heterodimers with the membrane-bound p22^phox^ subunit [64].

**Figure 1:**
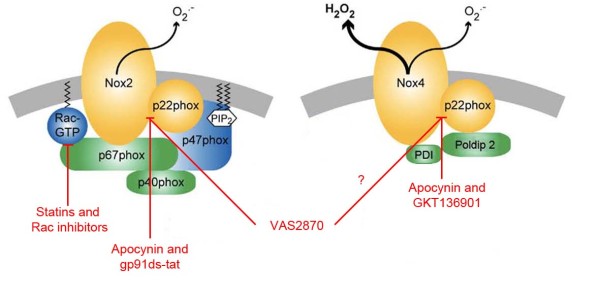
**Relevant NOX isoforms in stroke and their respective subunit requirements (adapted from**[46]**).** NOX2, as well as NOX4, seem to be implicated in stoke. Known regulatory proteins are associated with individual isoforms. Activator proteins are coloured in green and organizing proteins in blue. Both isoforms form functional dimers with p22^phox^. p47^phox^ phosphorylation subsequently causes the cytosolic subunits p47^phox^, p67^phox^, and p40^phox^ to translocate into membranes and fuse with the catalytic subunit NOX2. This is followed by interaction between Rac and NOX2. Nox4 forms a dimer with p22^phox^. Although NOX4 does not appear to require additional regulators, recently some NOX4 binding proteins (DPI and PolDip2) have been discovered whose role needs to be further elucidated. Potential target sites of NADPH oxidase inhibitors are also shown in the scheme.

NOX2 was the first isoform identified and thus is the best-studied isoform. Subunits that are needed for NOX2 activation are divided into two groups: activating molecules (p67^phox^) and organizing molecules (p47^phox^). Upon phosphorylation of p47^phox^, cytosolic subunits (p40^phox^, p47^phox^ and p67^phox^) translocate to the cell membrane and bind to intracellular loops of NOX2. Activators bind to Rac in the cytosol and then migrate towards NOX2 with help of the organizers [41] (Figure 1). For ROS production by NOX2, Rac and p47^phox^ have to be activated simultaneously.

Similar to NOX2, NOX1 is also regulated by cytosolic units. Its organizer molecule NOXO1 constitutively interacts with the membrane and does not need phosphorylation like p47^phox^. Therefore, NOX1 seems to be active under basal conditions [65]. The activating molecule of NOX1 is called NOXA1.

NOX4 seems to only need interaction with p22^phox^, but does not necessarily require any of the known cytosolic subunits to be active. NOX4 was therefore suggested to be constitutively active, and thus its activity may directly be related to its protein levels [66]. Recently, Rac implication in NOX4 activation has also been excluded [67]. However, stimulus-induced ROS production by NOX4 has been observed; and maybe other, yet unknown factors play a role in NOX4 activity regulation. It has been shown that polymerase (DNA-directed) delta interacting protein 2 (PolDip2) interacts with p22^phox^, which promotes NOX4 linking to the cytoskeleton [68] (Figure 1). Furthermore, protein disulphide isomerase (PDI) may also bind to NOX4, as well as to NOX1 [69]. However, the role of PolDip2 and PDI in regulating NADPH oxidases has to be further investigated.

NOX5 does not require heterodimerization with p22^phox^. Interestingly, NOX5 is directly activated by calcium, which can bind on the EF-hand motives of the N-terminal tail of NOX5 [41,46]. Although it is generally agreed that there is no need for other NOX5 binding proteins, a recent study showed that Hsp90 binds to NOX1, 2 and 5 and that this binding is needed to confer enzyme stability [70]. Five splice variants of NOX5 have been discovered, with the shortest isoform 5s lacking the calcium-binding domain [62].

Moreover, it was postulated that NADPH oxidases are involved in the ischemic pathway by functioning as oxygen sensors [71], an important aspect in the field of I/R injury. In hypoxic cells, mRNA and protein levels of NOX increased rapidly. The subsequent increase in ROS production led to an upregulated and activated hypoxia inducible factor (HIF) [31], whereas in NOX KO cells, HIF was not induced by hypoxia. Hypoxia seems to regulate all NOX isoforms [72-74].

Besides regulation through binding proteins, various stimuli, e.g. angiotensin II, thrombin or growth factors, have also been shown to alter the expression or the activity of NADPH oxidases [47]. NOX homologues can cause different physiological responses when coupled to different agonists.

### Enzymatic products

Reduction of oxygen by transmitting one electron from NADPH results in O_2_^-^ that can be converted into H_2_O_2_ by superoxide dismutases (SOD). All NOX isoforms appear to produce O_2_^-^, except NOX4 that mainly produces H_2_O_2_[55,75] (Figure 1). Because of this H_2_O_2_ production, initially several problems were encountered to detect ROS production from the NOX4 isoform [67,76]. Mutations of NOX4 can switch ROS release from H_2_O_2_ to O_2_^-^[75].

## Role of NADPH oxidases in stroke

In Table 1 we summarised the published pre-clinical stroke studies that investigated the role of NADPH oxidases for their adherence to some of the STAIR criteria. The first evidence of elevated or decreased NOX levels in a certain tissue is often provided by measuring mRNA levels. However, are mRNA levels really relevant for protein levels or even activity? Further, association of subunits plays a role in NOX activity. For NOX4, mRNA levels were suggested to be related to activity [77], but there are also reports that NOX4 is regulated at the translational level [78]. Generally, a better approach would be to assess protein levels and localisation. However, the major issue with detecting NOX proteins is the lack of specific and well-characterized antibodies.

To study the cause-effect relationship, NOX (or other subunit) overexpressing animals or KO mice are used [79]. Mice with a deleted NOX2 gene are commercially available. In this KO model, exon 3 of the NOX2 gene is deleted [80]. Two NOX1 KO models, lacking the same part of the NOX1 gene (exons 3–6), have been published by different groups [52,53]. Those mice showed a slightly hypotensive phenotype. Four different NOX4 KO have been published, and none of them shows a basal phenotype [12,58,81,82]. Other studies observed a reduced neuronal death in mice lacking the p47^phox^ subunit, which is required for NADPH oxidase assembly [83]. A problem in KO models is the possibility that a truncated NOX protein or an alternative splice variant with residual activity can still be formed when the deletion takes place in one of the early exons [84,85]. We therefore believe that it is a better approach to delete the exons coding for NADPH- or FAD-binding sites of the enzyme (exon 14–15 or exon 9 respectively).

An alternative approach in mice or other species is to use small interfering RNA (siRNA) to silence the gene of interest. When using this approach the isoform-specificity of the siRNA should be tested [86], and protein levels should be measured. However, specific antibodies would be needed.

Pharmacological NADPH oxidase inhibition presents another tool to validate the role of NOX in stroke (for review see [31,87]. Apocynin, diphenylene iodium (DPI) and 4-(2-aminoethyl)-benzensulfonylfluorid (AEBSF) have shown neuroprotective potential in vivo [88-90]. However, these drugs are not specific to NADPH oxidase inhibition. DPI blocks enzymatic flavin sites in general [91] and AEBSF is a non-specific serine protease inhibitor [92]. For apocynin activation by neutrophil-secreted myeloperoxidase is required, which is not present in some cell types such as VSMCs [93]. Further, apocynin has antioxidant properties [93] and also inhibits rho kinase [94,95]. Rho kinase-inhibition could be the actual neuroprotective mechanism of apocynin as Rho kinase is implicated in stroke [96]. Therefore, any results on the role of NADPH oxidase obtained by using apocyin should be questioned. Besides, the therapeutic window of apocynin is quite narrow, and doses above 2,5 mg/kg actually increased cerebral hemorrhage and post-stroke mortality [17,97]. Even so, it is the most frequently used compound to provide NADPH oxidase inhibition in stroke studies. It was reported to reduce post-ischemic ROS production in neurons and microglia [98,99] and showed neuroprotective effects in stroke [15,88]. In most studies, apocyin was given prior to stroke onset, which is not reflecting the clinical situation. Apocynin was found to reduce cerebral ROS levels when administered pre-stoke but not post-stroke [17]. Similarly, when given post-stroke, Kleinschnitz et al. observed no effect of apocynin on stroke outcomes in mice [12].

A more specific NOX inhibitor, VAS2870 and its derivative VAS3947, likely inhibit all NOX isoforms. These compounds were shown to be free of general flavin protein inhibition or ROS scavenging activities [100]. Importantly, when given hours after stroke, less ROS production, reduced infarct volume and better neurological function were observed in VAS2870 treated WT mice [12]. However, just recently off-target effects of VAS2870 have been published. As no molecular mechanism has been proposed for NOX inhibition by VAS2870, authors suggest the possibility that Cys thiol alkylation may play a role in NOX4 inhibition [101].

Another specific inhibitor is gp91ds-tat, a peptide that blocks the p47^phox^ binding site of NOX2, thereby inhibiting its activity. Possibly also the activity of NOX1 is inhibited but an effect on NOX4 and NOX5 is unlikely [102]. Recently, GKT136901 was introduced as a highly potent NADPH oxidase inhibitor with dual activity on NOX1 and NOX4 [103]. However, neither data using gp91ds-tat nor GKT136901 in stroke studies have been published.

## NOX studies performed in the stroke field

Most data derive from RT-PCR studies, as protein expressions are often very low and specific antibodies difficult to obtain. mRNA and even protein levels of NOX1, 2, 4 and 5 were discovered in healthy brains of mice, rats and humans with NOX2 and 4 being the dominant isoforms [104]. This presence suggests a physiological role. Activities of NADPH oxidases are even higher in rat and mouse cerebral arteries versus systemic vessels [105].

### NOX1

In cell cultures of the murine brain, NOX1 protein was found in neurons, astrocytes, microglia and endothelial cells [11]. Results on the role of NOX1 in stroke using NOX1 KO mice are conflicting. Jackman et al. [10] did not observe an effect of NOX1 deletion on total infarct volume, edema or neurological outcome after tMCAO when compared to WT mice. However, they did find an increased cortical infarct in NOX1 KOs, suggesting that NOX1 might play a role in limiting cortical infarct size. Basal ROS levels in the brains were similar in WT and NOX1 KO mice. A second group [11] observed the opposite effects of NOX1 KO on stroke. They reported a 55% smaller stroke lesion that was paralleled by better neurological outcome in NOX1 KO versus littermate WT mice after 1 h of ischemia and 23 h of reperfusion. However, no difference in lesion size was observed when the ischemic period lasted 2 h or when permanent ischemia was investigated. Apoptosis levels were similar in both strains and the response to antioxidants and NOS inhibitors was similar in both genotypes. Thus, the observed infarct size reduction in NOX1 KO mice was neither related to reduced ROS production nor to reduced NO bioavailability. A third study reported no significant difference in stroke outcome between NOX1 deficient and control mice 1 day after cerebral I/R injury [12].

In summary, it is unlikely that NOX1 plays a major role in stroke in mice. One should also keep in mind that NOX1- and p47phox deficient mice had reduced basal blood pressures and blunted pressor responses to angiotensin II [52,53,106], which may interfere with stroke outcome.

### NOX 2

**Figure 2:**
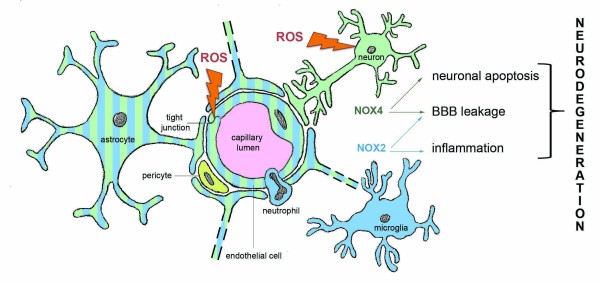
**Schematic overview of NADPH oxidases implicated in stroke.** The blood–brain barrier (BBB) is formed by endothelial cells at the level of the cerebral capillaries. The figure shows a brain capillary in cross section, showing endothelial tight junctions and end-feet of astrocytes covering these capillaries. The figure also shows pericytes, neurons and microglial cells. Cerebral NOX activation and subsequent ROS generation contributes to BBB disruption, inflammation and postischemic neuronal injury.

The most studied isoform in stroke experiments is NOX2. After transient focal cerebral ischemia NOX2 protein levels were elevated, p47^phox^ translocated to the membrane and ROS production increased [107]. These results show a potential role of NOX2 in stroke pathology (Figure 2). The spatiotemporal profile of NOX2 expression was studied in endothelin-1-induced stroke in conscious rats [108]. NOX2 mRNA was upregulated from 6 hours until 7 days post-stroke in the cortex and striatum. At 6 h post-stroke ROS production was found in neurons, and after 7 days in macrophages and microglia.

Several papers reported a protective effect of deleting NOX2 in mice: NOX2 deficient mice had a 40% smaller infarct volume than WT mice [13-21]. In contrast to these studies, Kleinschnitz and colleagues could not reproduce the findings of the previous studies concerning the neuroprotective effect of NOX2 deletion despite using a high number of animals (n = 19) [12]. VAS2870 did not display an additional protective effect in NOX4 knockout mice, whereas it did so in WT mice, further suggesting that NOX1 and 2 have no major implication in stroke pathology [12]. Reasons for the discrepancies are unclear, but several factors may play a role, including different occlusion times and different experimental protocols, as discussed in more detail in the review of Radermacher et al. (Antioxid Redox Signal, in revision).

### NOX4

**Figure 3:**
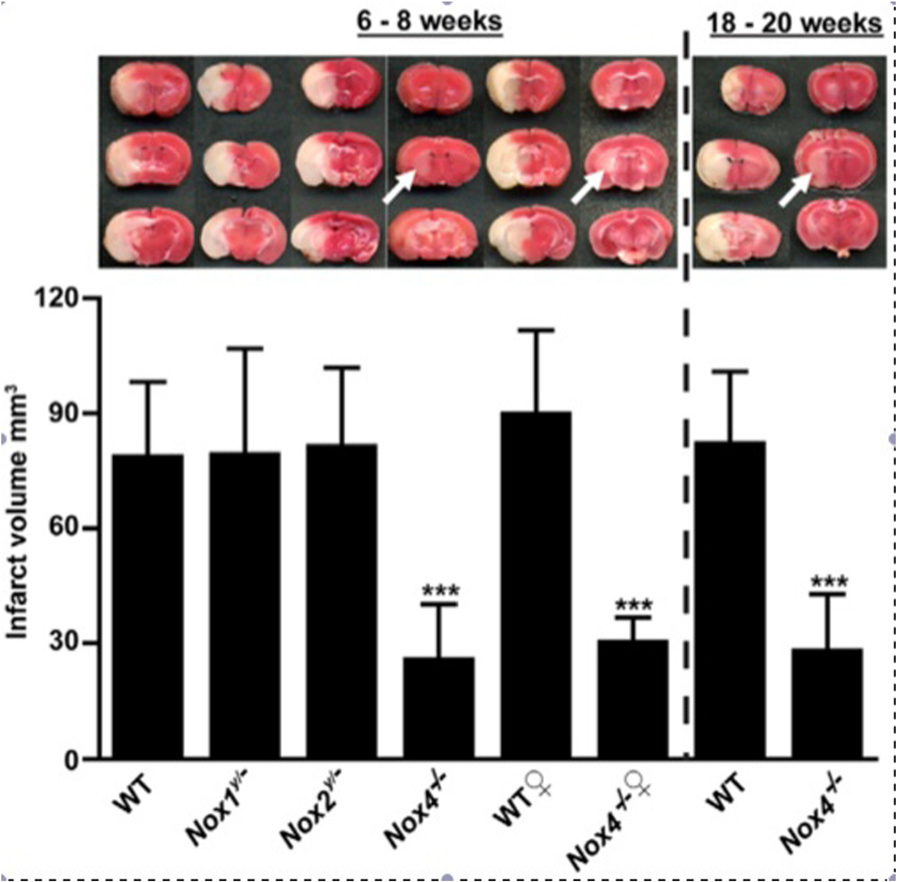
**NOX4 deletion confers neuroprotection during stroke**[12]**.** The upper images show the TTC staining of coronal brain slices after cerebral I/R in WT, NOX1-, NOX2- and NOX4 KO mice on 24 hours after tMCAO (1 hour ischemia). The infarct volume (white region) is about 75% smaller in NOX4 deficient mice compared to the other mice, as also illustrated by the bar graph. Stroke experiments were also performed in female mice and in older animals, obtaining the same results.

In the brain, NOX4 is most abundant in endothelial cells, but also present in neurons and astrocytes (Figure 2) [11]. As for NOX2, the spatiotemporal profile of NOX4 mRNA expression after stroke in rats has been measured [108]. NOX4 mRNA was upregulated 6 h post-stroke and then returned to control levels. In contrast, others reported elevated NOX4 mRNA levels in the rat cortex 24 h after pMCAO lasting up to 15 days. The early elevation of NOX4 mRNA is probably due to neuronal expression and the later peak expression to neo-angiogenesis [60]. In another study, NOX4 mRNA was elevated at 12 and 24 h after tMCAO [12]. Here, NOX4 expression was also validated at the protein level (by immunohistochemistry) in brain samples from stroke patients and in mouse brain slices. Co-staining clearly showed colocalization of NOX4 with endothelial cells and neurons. To our knowledge only one study performed stroke experiments in NOX4 KO mice [12] (Figure 3). After exclusion of systemic vascular effects in NOX4 deficient mice, 75% smaller infarct volumes were measured in NOX4 KO compared to WT, NOX1 KO and NOX2 KO mice (Figure 3). In addition, functional outcomes were improved, as was survival. Similar results were obtained in female, in older mice (18–20 weeks) and when using a permanent stroke model. This study shows that NOX4 seems to play a major role in brain damage and thus is a promising target in stroke therapy. In addition to this NOX4 KO study, another group recently showed first results confirming the detrimental role of NOX4 post stroke. For their study, this group used transgenic mice that overexpress NOX4 in endothelial cells. One day after MCAO, the infarcted region of these transgenic animals was bigger than in wildtype mice and suppression of eNOS by NOX4 was proposed as being responsible for infarct enlargement [109].

### NOX5

As NOX5 is not expressed in rats and mice, studies investigating this isoform are scarce, and thus to date no data on the role of NOX5 in stroke are published. To elucidate the possible role of this isoform in animals, transgenic expression of NOX5 in mice is needed, or other animal species, such as rabbits, have to be used.

## Summary

Excessive NADPH oxidase-derived ROS are likely to lead to neurodegeneration via breakdown of the blood–brain-barrier and/or via neuronal apoptosis (Figure 2). However, ROS-scavenging antioxidants have shown disappointing results in clinical trials. Stroke research should move on and tackle oxidative stress at its root, meaning specifically inhibiting the disease-relevant source of ROS, such as NADPH oxidases, rather than attempting to detoxify them in an untargeted fashion after they have been formed. When neuroprotection during stroke was studied, a maximal reduction in infarct volume of 30-40% was achieved in NOX2 deficient mice [110]. However, in NOX4 KO mice, bigger changes (75%) could be observed [12]. Furthermore, in NOX4 KO mice no basal phenotype has been observed yet, suggesting that side effects from therapeutic inhibition of NOX4 should be rather small.

## Conclusions

Until now, the cellular and molecular origin of ROS causing I/R injury in the brain has not been fully identified yet. However, NADPH oxidases are promising therapeutic targets for stroke therapy. Stroke therapy has a very short therapeutic window; the drug has to be administered quite soon after stroke onset to avoid neuronal death. If NOX inhibitors turn out to be promising therapeutic agents, the optimal therapeutic window has to be established and the drug will probably have to be tested in presence of rt-PA that will still be used for initial clot lysis.

Modern neuroimaging techniques are now available, particularly magnetic resonance imaging and angiography of the brain [111-113]. These tools should also be used in pre-clinical studies to monitor long-term outcome and to show that the treatment not only alters recovery kinetics, but really provides long-term protection [114-116]. Only then it can be ruled out that the evolution of stroke is just slowed down rather than stopped.

Despite recent progress, much remains to be learned about NADPH oxidases as potential pathological sources of ROS production. Therefore, for instance, conditional KO models in specific cell types are warranted and more studies are particularly needed on the non-rodent isoform NOX5. As studies on the role of NADPH oxidases in stroke so far focussed on mice or rats, other species than rodents could be useful for clinical translation.

Excitingly, NADPH oxidases are not only implicated in stroke, they may also serve as novel therapeutic target for other cardiovascular [117] and neurodegenerative diseases [118]. As such, NOX inhibitors could thus serve as powerful therapeutic strategy in pathologies where oxidative stress is implicated. Clearly, isoform-selective NOX inhibitors would help to establish the role of the different isoforms in diseases and to successfully translate this novel strategy into the clinic. This would be a major breakthrough after years without any major therapeutic progress in stroke therapy.

## Abbreviations

AEBSF: 4-(2-aminoethyl)-benzensulfonylfluorid; AO: Antioxidant; BBB: Blood brain barrier; cDNA: Complementary deoxyribonucleic acid; CGD: Chronic granulomatous disease; CNS: Central nervous system; COX: Cyclooxygenase; CYP450: Cytochrome P450; DHE: Dihydroethidium; DPI: Diphenylene iodonium; DUOX: Dual oxidase; ECs: Endothelial cells; ER: Endoplasmic reticulum; FAD: Flavin adenine dinucleotide; FDA: Food and Drug Administration; H2O2: Hydrogen peroxide; HIF: Hypoxia inducible factor; Hsp90: Heat shock protein 90; I/R: Ischemia-reperfusion; KO: Knockout; MCAO: Middle cerebral artery occlusion; NADPH: Nicotinamide adenine dinucleotide phosphate; NO: Nitric oxide; NOS: Nitric oxide synthase; NOX: catalytic subunit of NADPH oxidase; n.s.: not specified; O2-: Superoxide; OONO-: Peroxynitrite; PCR: Polymerase chain reaction; PDI: Protein disulphide isomerase; PolDip2: Polymerase (DNA-directed) delta interacting protein; RNS: Reactive nitrogen species; ROS: Reactive oxygen species; siRNA: Small interfering ribonucleic acid; SOD: Superoxide dismutase; STAIR: Stroke Therapy Academic Industry Roundtable; rt-PA: Recombinant tissue plasminogen activator; TTC: 2,3,5-triphenyltetrazolium chloride; VSMCs: Vascular smooth muscle cells; XO: Xanthine oxidase.

## Competing interests

Harald Schmidt has received funding from Servier to conduct research on NADPH oxidase and has founded and holds shares in Vasopharm GmbH, a biotech company engaged in NOX inhibitor development. Christoph Kleinschnitz has received funding from Novartis Pharma GmbH, Nürnberg, Germany to conduct research on NADPH oxidase. Kirstin Wingler is a former employee of Vasopharm GmbH.

## Authors’ contributions

The authors have made the following declaration about their contributions: Conceived and gave final approval of the manuscript: HHHWS. Wrote draft of the paper: KAR. Contributed to the writing by critically revising and proof reading of the manuscript: KW, SA, PK, JJRH, CK. All authors read and approved the final manuscript.
